# Consumption of a Low Carbohydrate Diet in Overweight or Obese Pregnant Women Is Associated with Longer Gestation of Pregnancy

**DOI:** 10.3390/nu13103511

**Published:** 2021-10-05

**Authors:** Helen Tanner, Helen L. Barrett, Leonie K. Callaway, Shelley A. Wilkinson, Marloes Dekker Nitert

**Affiliations:** 1 Department of Obstetric Medicine, Royal Brisbane and Women’s Hospital, Metro North Hospital and Health Service, Brisbane, QLD 4029, Australia; Leonie.Callaway@health.qld.gov.au; 2 School of Clinical Medicine, University of Queensland, Brisbane, QLD 4029, Australia; 3 Mater Research Institute, University of Queensland, Brisbane, QLD 4029, Australia; Helen.Barrett@mater.uq.edu.au; 4 Department of Endocrinology, Mater Hospital, Brisbane, QLD 4029, Australia; 5 School of Human Movement and Nutrition Sciences, Faculty of Health and Behavioural Sciences, University of Queensland, Brisbane, QLD 4072, Australia; s.wilkinson@uq.edu.au; 6 School of Chemistry and Molecular Biosciences, University of Queensland, Brisbane 4072, QLD, Australia; m.dekker@uq.edu.au

**Keywords:** pregnancy, carbohydrate, ketones, gestation, birth centile, diet, macronutrient

## Abstract

Studies of obstetric outcomes in women consuming low-carbohydrate diets have reported conflicting results. Most studies have defined low-carbohydrate diets by the percentage that carbohydrates contribute to overall energy intake, rather than by an absolute amount in grams per day (g/d). We hypothesised that a low absolute carbohydrate diet affects obstetric outcomes differently than a low percentage carbohydrate diet. Dietary data were collected from overweight or obese women in the Study of Probiotic IN Gestational diabetes at 16- and 28-weeks’ gestation. Obstetric outcomes were compared between women whose carbohydrate intake was in the lowest quintile vs quintiles 2–5. Mean gestation was increased in women whose absolute carbohydrate intake was in the lowest quintile at 16 and at both 16- and 28-weeks’ gestation compared with all other women (16: 39.7 vs. 39.1 weeks, *p* = 0.008; 16 and 28: 39.8 vs. 39.1, *p* = 0.005). In linear regression analysis, a low absolute carbohydrate intake at 16 and at 28 weeks’ gestation was associated with increased gestation at delivery (16: *p* = 0.04, adjusted R^2^ = 0.15, 28: *p* = 0.04, adjusted R^2^ = 0.17). The coefficient of beta at 16 weeks’ gestation was 0.50 (95% CI 0.03–0.98) and at 28 weeks’ gestation was 0.51 (95%CI 0.03–0.99) meaning that consumption of a low absolute carbohydrate diet accounted for an extra 3.5 days in gestational age. This finding was not seen in women whose percentage carbohydrate intake was in the lowest quintile. Low-carbohydrate consumption in pregnancy is associated with increased gestational age at delivery.

## 1. Introduction

Studies evaluating obstetric outcomes in women consuming low-carbohydrate diets in pregnancy have reported conflicting results [1,2,3]. In most studies, low carbohydrate diets are defined by the carbohydrate contribution to overall energy intake, usually less than 40–45% of total energy [1,2,3]. Low carbohydrate diets can precipitate ketone production (ketogenesis) and therefore have the potential to change the maternal metabolic environment. However, ‘ketogenic diets’ are generally defined by the carbohydrate intake in grams per day (g/d) rather than by the percentage that carbohydrate contributes to overall kilojoule (kJ) intake.

The exact amount of carbohydrate required to avoid increased ketogenesis in pregnancy is unknown. In the non-pregnant population, a carbohydrate intake of less than 50 g/d is generally considered to be ketogenic [4]. Ketogenesis is accelerated in pregnancy, implying that it is possible that an amount greater than 50 g/d of carbohydrates will still result in increased ketone production [5]. Current guidelines advise pregnant women to consume a minimum of 175 g/d of carbohydrate [6]. However, this advice is not based on evidence that consumption of less than this amount increases maternal ketone levels. Consumption of 165 g/d of carbohydrates in a small group of women with GDM, did not result in elevated fasting levels of the ketone beta-hydroxybutyrate (BHB), suggesting that carbohydrate intake needs to be lower than 165 g/d to increase ketone production [7].

A diet that is sufficiently low in absolute carbohydrate intake, such that ketogenesis is increased, may have a different effect on pregnancy outcomes from a diet where there is a low percentage of kJ from carbohydrates. To evaluate this, we analysed dietary carbohydrate intake in a cohort of overweight and obese pregnant women. Obstetric outcomes were evaluated in women who consumed a diet with carbohydrate intake in the lowest quintile as measured in g/d, compared with all other women. As a comparison, obstetric outcomes were evaluated in women who consumed a diet with carbohydrate intake in the lowest quintile, as measured by the percentage that carbohydrate contributed to overall energy intake, compared with all other women. 

## 2. Materials and Methods

Dietary questionnaires were completed by women enrolled in the Study of Probiotic IN Gestational diabetes (SPRING), a study of probiotics to prevent GDM in overweight and obese pregnant women [8]. Daily carbohydrate intake was assessed at 16- and 28-weeks’ gestation using the validated Cancer Council Victoria’s Dietary Questionnaire for Epidemiological Studies V2.0 [9,10]. 

**Figure 1:**
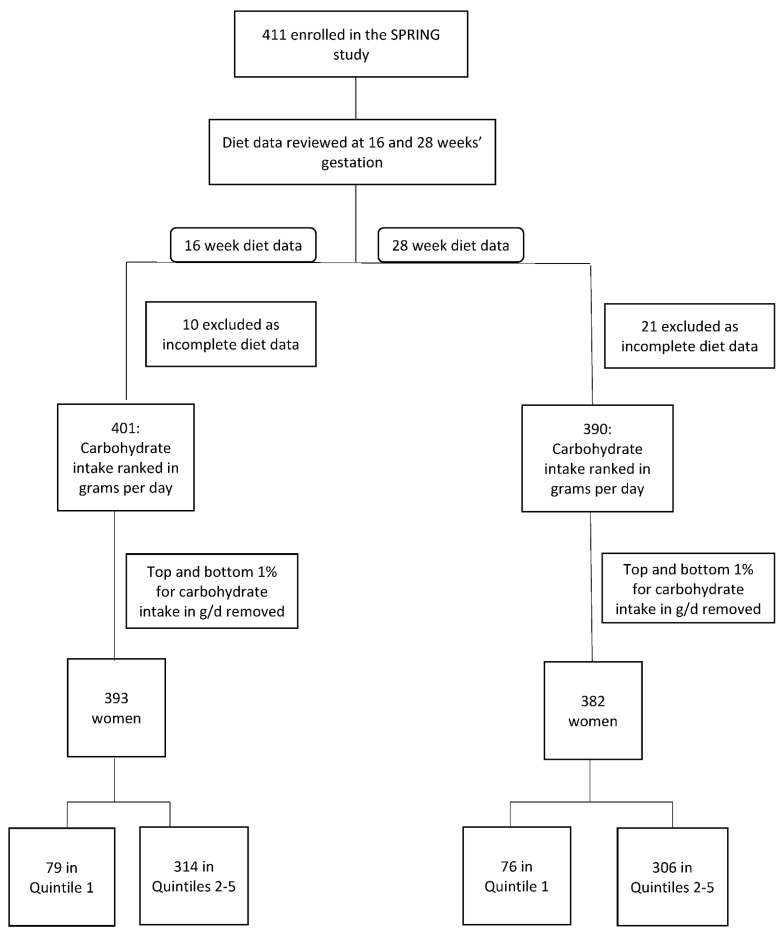
Participant Flowchart.

Carbohydrate intake was analysed by quintiles for both absolute carbohydrate intake in g/d and the percentage that carbohydrate contributed to overall energy intake. A diet where carbohydrate intake measured in g/d was in the lowest quintile was defined as a low absolute carbohydrate diet (LaCD) and a diet where the percentage of carbohydrate in the diet was in the lowest quintile was defined as a low percentage carbohydrate diet (LpCD). A diet with carbohydrate intake in g/d in quintiles 2–5 was deemed to be a standard diet (SD) and a diet where the percentage of carbohydrate was in quintiles 2–5 was deemed to be a standard percentage diet (SPD). Women consuming a diet where carbohydrate intake was in the lowest quintile were compared with women consuming a diet where carbohydrate intake was in quintiles 2–5, such that women consuming the lowest amount of carbohydrate were compared with all others. Outliers were defined as participants with a carbohydrate intake in g/d in the top and bottom 1% of participants and were removed from the analysis to account for over- and under-reporting (Figure 1).

Demographic data were collected on all patients including ethnicity, maternal age at delivery, body mass index based on measured weight at 16 weeks’ gestation (BMI), parity, intervention group in the study (probiotic or placebo), previous personal history of GDM and immediate family member diagnosed with diabetes (family history of diabetes). Dietary intake was analysed at both 16 (baseline) and 28 weeks’ gestation and included total daily kilojoule (kJ), carbohydrate, fat, protein, fibre and junk food intake. Junk food was defined as per the Junk Food Index [11]. Dietary micronutrient intake was analysed for the carbohydrate-specific nutrients folate and thiamine. This did not include micronutrient intake from multivitamin supplements. Fasting metabolic parameters were obtained at both 16- and 28-weeks’ gestation including blood glucose (FBG), c-peptide, insulin, triglyceride and cholesterol levels. Insulin resistance (HOMA-IR) was calculated using FBG and insulin levels. 

The following obstetric outcomes were obtained for each woman: birth weight, birth weight z score, birth centile, low birth weight (LBW, birthweight < 2.5 kg), macrosomia (birth weight > 4.0 kg), preeclampsia (PET) defined as per the Society of Obstetric Medicine of Australia and New Zealand (SOMANZ) hypertension guidelines [12], hypertensive disorder of pregnancy (HDP), gestational diabetes mellitus (GDM) status defined as per the International Association of the Diabetes and Pregnancy Study Group (IADPSG) criteria [13], induction of labour (IOL) and/or caesarean section (C-section) (these were combined to account for all deliveries that were not spontaneous), gestational age at delivery, pre-term delivery (34) (<34 weeks), pre-term delivery (37) (<37 weeks, including babies born prior to 34 weeks’ gestation), full-term delivery (≥40 weeks), gestational weight gain (self-report in early pregnancy to measured weight at 36 weeks’ gestation), neonatal hypoglycaemia (<2.5 mmol/L), neonatal intensive care nursery (ICN) admission, neonatal jaundice and neonatal respiratory difficulties. Birth centile was calculated using the Perinatal Institute global bulk centile calculator (BCC version 8.0.6.1, 2020). The factors included in this calculator are ethnicity, maternal BMI, parity, infant sex, gestation at delivery, birthweight and live birth outcome. Birth weight z scores were calculated using Australian birth weight data [14].

Categorical variables are reported as number and percentage (%). Continuous variables are reported as mean and standard deviations (SD). 

## 3. Statistics

### 3.1. Dietary and Metabolic Analysis

T-tests were performed to assess for significant differences in dietary intake and metabolic parameters at 16- and 28-weeks’ gestation between women consuming a LaCD and SD at 16- and 28-weeks’ gestation respectively, and between women consuming a LpCD and SPD at 16- and 28-weeks’ gestation respectively.

### 3.2. Pregnancy Outcomes

The effect of a low carbohydrate diet on obstetric outcomes was examined in three ways; (1) comparing outcomes in women consuming a LaCD with women consuming a SD at 16 weeks’ gestation; (2) comparing outcomes in women consuming a LaCD with women consuming a SD at 28 weeks’ gestation; and (3) comparing outcomes in women consuming a LaCD diet at both 16 and 28 weeks’ gestation with women consuming a SD at *both* 16- and 28-weeks’ gestation. This final comparison excluded women consuming a LaCD at only one time point. The same analyses were performed comparing outcomes in women consuming a LpCD and an SPD. 

To control for the effect of confounders on the length of gestation and birth centile in women consuming a LaCD, linear regression was performed. The following demographic and obstetric factors were initially examined in a univariate analysis: maternal age at delivery, parity, BMI, ethnicity, probiotic use, family history of diabetes, GDM status, IOL and/or C-section, hypertensive disorder of pregnancy, infant sex and weight gain during the pregnancy. All factors that reached a significance level of *p* < 0.1 were entered into a multivariate linear regression model. Results were considered statistically significant if *p* < 0.05. 

In addition to the analysis of carbohydrate intake by quintiles, obstetric outcomes were compared between women consuming greater or less than the currently recommended minimum intake of 175 g/d of carbohydrate at 16- and 28-weeks’ gestation. 

Fisher’s exact tests were used to compare groups for significant differences in the rates of obstetric outcomes. *T*-tests were used to assess for statistical differences in mean outcomes between groups. The level of significance used was *p* < 0.05. When comparing for differences in obstetric outcomes between groups, an adjusted *p* value of <0.003 was also used to account for multiple comparisons. Data were analysed with R, version 3.1.2. Ethics approval was granted by the Royal Brisbane and Women’s Hospital Ethics Committee (HREC/11/QRBW/467). All participants gave informed consent prior to enrolment in the SPRING study. 

## 4. Results

Four hundred and eleven women were enrolled in the SPRING study. Ten women were removed from the 16-week analysis and 21 from the 28-week analysis due to missing dietary data. Eight women were removed as outliers (Figure 1).

Patient characteristics for women consuming a LaCD and a SD are shown in Table 1. Participants are divided into three groups: LaCD vs. SD at 16 weeks’ gestation, LaCD vs. SD at 28 weeks’ gestation, and LaCD vs. SD at both 16- and 28-weeks’ gestation. Patient characteristics for women consuming a LpCD and a SPD are shown in Supplementary Table S1. Participants are divided into three groups: LpCD vs. SPD at 16 weeks’ gestation, LpCD vs. SPD at 28 weeks’ gestation, and LpCD vs. SPD at both 16- and 28-weeks’ gestation.

Dietary intake and metabolic parameters for women consuming a LaCD and a SD are shown in Table 2 and for LpCD and SPD in Supplementary Table S2. There was significant crossover between groups with many women consuming a diet that met the definition for both a LaCD and a LpCD.

### 4.1. Obstetric Outcomes

#### 4.1.1. Consumption of Low Absolute Carbohydrate Diet (LaCD) vs. a Standard Diet (SD)

Mean gestation of pregnancy was longer in women consuming a LaCD compared with a SD at 16 and at 16- and 28-weeks’ gestation, but not at 28 weeks’ gestation. (16: 39.7 vs. 39.1 weeks, *p* = 0.008; 28: 39.5 vs. 39.2 weeks, *p* = 0.20; 16 and 28: 39.8 vs. 39.1, *p* = 0.005) (Table 3). The power for detecting a difference in gestation at delivery was 84.7%. Mean birth centile was lower in women consuming a LaCD compared with a SD at 28 weeks’ gestation and at 16- and 28-weeks’ gestation, but not at 16 weeks’ gestation (16: 46th v 52nd, *p* = 0.08; 28: 45th vs. 53rd centile, *p* = 0.04; 16 and 28: 43rd vs. 53rd centile, *p* = 0.04). No other statistically significant differences in obstetric outcomes were found between women consuming a LaCD and a SD. When adjusting the *p*-value cut-off for the number of comparisons performed (*p*-value cut-off = 0.003), the gestational age at delivery analysis trended to be close to significant but the birth centile associations did not.

#### 4.1.2. Sensitivity Analysis

Due to the potential confounder of GDM on both carbohydrate intake and gestation at delivery and birth centile, we performed sensitivity analysis by removing all participants who developed GDM. After removing these women, gestation at delivery remained increased in women consuming a LaCD at 16 and at both 16 and 28 weeks compared with a SD (16: 39.9 vs. 39.4, *p* = 0.004; 16&28: 40.0 vs. 39.4, *p* = 0.004). Birth centile was also reduced in women consuming a LaCD at 16, 28 and at both 16- and 28-weeks’ gestation compared with a SD (16: 43.5 vs. 53.4, *p* = 0.02; 28: 42.0 vs. 53.8, *p* = 0.005; 16&28 38.9 vs. 54.1, *p* = 0.003). 

#### 4.1.3. Linear Regression

The following factors were found to be associated with gestation at delivery when performing univariate analysis using a *p* < 0.1; BMI, previous GDM, infant sex, GDM, HDP, IOL or C-section and gestational weight gain. After adjusting for these factors, consumption of a LaCD at 16 and at 28 weeks’ gestation was significantly associated with increased gestation at delivery when analysed via linear regression: (16: *p* = 0.04, adjusted R^2^ = 0.15, 28: *p* = 0.04, adjusted R^2^ = 0.17). The coefficient of beta for consumption of a LaCD at 16 weeks’ gestation was 0.50 (95% CI 0.03–0.98) and at 28 weeks’ gestation was 0.51 (95%CI 0.03–0.99) meaning that consumption of a LaCD accounted for an extra 3.5 days in gestational age. All other factors that were significantly associated with gestation in the model reduced the gestational age at delivery. 

The following factors were found to be associated with birth centile when performing univariate analysis using a *p* < 0.1; family history of diabetes and gestational weight gain. After adjusting for these factors, there was a trend to an association between the consumption of a LaCD at 16 weeks and at 28 weeks’ gestation and birth centile (16: *p* = 0.08, adjusted R^2^ = 0.02, 28: *p* = 0.07 adjusted R^2^ = 0.02).

### 4.2. Consumption of a LpCD vs. a SPD

Gestational weight gain was significantly reduced in women consuming a LpCD compared with women consuming an SPD at 28 weeks’ gestation only (6.9 vs. 8.2 kg, *p* = 0.04) despite similar energy intake (Supplementary Table S3). The rate of neonatal hypoglycemia was significantly increased in women consuming a LpCD compared with an SPD at 28 weeks’ gestation only (21% vs. 11%, *p* = 0.04). No other statistically significant differences in obstetric outcomes were found. In particular, consumption of a LpCD was not associated with increased gestation or reduced birth centile.

### 4.3. Association between Other Macronutrients and Gestation at Delivery and Birth Centile 

Total energy, fat and protein intake were analysed by quintiles to determine if these factors were associated with gestation at delivery or with birth centile. Mean gestation was increased in women whose total energy intake (kJ/d) and protein intake (g/d) were in the lowest quintile at 16 weeks’ gestation (kJ: 39.6 v 39.1, *p* = 0.009; protein 39.6 vs. 39.1, *p* = 0.02) (Table 4). Fat intake (g/d) at 16 weeks’ gestation was not associated with increased gestation. There was no association between total energy, fat or protein intake at 28 weeks’ gestation and gestation at delivery. There was also no association between total energy, fat or protein intake at either 16- or 28-weeks’ gestation and birth centile.

### 4.4. Consumption of Greater or Less Than 175 g/d of Carbohydrate

There were no significant differences in obstetric outcomes between women consuming less than 175 g/d of carbohydrate at 16 weeks’ gestation compared with women consuming greater than 175 g/d. However, rates of hypertensive disorders of pregnancy, IOL or C-section and neonatal jaundice were significantly lower amongst women consuming less than 175 g/d of carbohydrate at 28 weeks’ gestation (Table 5).

## 5. Discussion

We found that in overweight and obese pregnant women, a LaCD was associated with increased gestation at delivery and there was a trend towards reduced birth centile. The adjusted R^2^ value for the association between a LaCD and gestation at delivery was 0.15 at 16 weeks’ and 0.17 at 28 weeks’ gestation, indicating that consumption of a LaCD explained 15–17% of the variability in gestation at delivery. The results for the coefficient of beta showed that consumption of a LaCD accounted for an additional 3.5 days in gestational age at delivery. Women consuming a LaCD also consumed significantly less absolute protein, fat, and overall kJ compared with women consuming a SD. Low overall energy and protein intake at 16 weeks’ gestation were also associated with increased gestation at delivery, suggesting that gestation is increased in women with lower overall food intake. Previous research in a small cohort found similar results with both carbohydrate intake (g/d per patient weight in kilograms) and overall energy intake between 17 and 27 weeks’ gestation being significantly inversely associated with gestation of delivery (carbohydrate: r = −0.131, *p* < 0.05, energy intake: r = −0.133, *p* < 0.05) [15]. Neither fat nor protein intake in this gestational time period were associated with gestation at delivery. Birth centile was not reported in this study. 

Gestation at delivery and birth centile were not different between women consuming a LpCD and a SPD. This is despite there also being a significant difference in carbohydrate consumption in g/d between these women. However, carbohydrate intake in g/d amongst women consuming a LpCD was significantly greater than in women consuming a LaCD (16 weeks: 139 vs. 99 g, 28 weeks: 101 vs. 142 g). Potentially there is a change in the maternal metabolic environment that occurs when women consume less than 100 g/d of carbohydrates, and it is this change that is associated with increased gestation at delivery and reduced birth centile. Energy intake may also be playing a role as overall kJ intake, which was significantly lower in women consuming a low absolute but not low percentage carbohydrate diet, was also associated with increased gestation at delivery. There is little data in the literature to explain these findings. Potential theories could include that a low carbohydrate and overall energy intake leads to reduced fetal growth which in turn leads to increased gestation at delivery. Alternatively, a low carbohydrate, low energy diet is highly likely to result in increased ketone levels. Given that ketones are incorporated into cerebral lipids and proteins in the brain, it is possible that a ketogenic environment is advantageous to the developing fetus and for this reason it remains in utero for longer [16]. It could also be speculated that low carbohydrate and energy supply indicate that food is scarce outside the materno-fetal unit, and in this environment, the fetus is programmed to remain in utero for longer. More research would be required to determine whether the associations seen in this study are reproducible in larger datasets and to determine the biological reason for the association. 

Intake of dietary folate and thiamine was particularly low in women consuming a LaCD. The recommended daily intake for folate in pregnancy is 600 mcg and for thiamine is 1.4 mg [17]. In our study, women consuming a LaCD consumed 140–160 mcg of folate and 0.8 mg of thiamine from food per day. Around 50% of Australia’s thiamine intake comes from cereals and grains [18] and folic acid has been added to flour for breadmaking since 2009 to address population-wide deficiencies and the effects of these on fetal development [19]. Our finding identifies a potential concern with LaCDs in pregnancy and highlights the importance of extra micronutrient supplementation with multivitamins in women who choose to consume such diets. Fibre intake was also low in women consuming a LaCD. Recommended fibre intake is 28 g/d and the average fibre intake amongst these women was 11–13 g/d [17]. The effect of low fibre intake in pregnancy is not well understood; however, studies have shown that low fibre diets are associated with changes in the gut microbiome in pregnancy [20].

Gestational weight gain in pregnancy was significantly reduced in women consuming a LpCD at 28 weeks’ gestation compared with women consuming an SPD. Overall energy intake was not different between these two groups of women. This finding has not been previously seen in trials of low-carbohydrate diets for the treatment of GDM [1,2,3]. Interestingly, in our study, no difference was seen in gestational weight gain in women consuming a LaCD, despite lower overall energy intake in these women. This may reflect differing metabolic rates in these women or may also represent under-reporting in the food frequency questionnaire. 

The rates of hypertensive disorders of pregnancy, IOL or C-section and neonatal jaundice were lower amongst women consuming less than the recommended carbohydrate intake of 175 g/d when using a *p*-value of 0.05, however this became non-significant when adjusting for multiple comparisons. This is still an interesting finding and raises questions about the rationale for the current recommendation for minimum carbohydrate intake in pregnancy. However, this finding needs to be interpreted with caution; output from food frequency questionnaires provides relative and ranked values, rather than being a specific, absolute measure of intake [21]. 

While the increase in gestation seen in this study is fairly small and at the latter end of pregnancy, the finding is of clinical importance. The risk of special education needs is 1.16 times higher in children born at 37–39 weeks’ gestation compared with children born at 40 weeks’ gestation [22]. In a meta-analysis, children born at 37–38 weeks’ gestation were also found to have lower childhood IQ scores compared with those born at 39–41 weeks’ gestation [23]. However, the first study did not account for socio-economic status and not all studies in the meta-analysis accounted for this either. Neither study undertook sibling analysis. In a study that did perform sibling analysis by restricting the analysis to comparisons between siblings born at different gestational ages, no association was found between preterm birth and school performance [24]. Interestingly, a diet with low carbohydrate and overall kJ intake has the potential to lead to increased ketone production. Some studies have found an association between maternal ketones and reduced childhood IQ; however, studies are conflicting and the relationship between maternal ketones and childhood IQ is uncertain [25]. 

Further research needs to be done to explore our findings in more detail. Gestational age at delivery and birth centile should be evaluated in a larger group of women consuming a LaCD with measurement of serum ketones. The association between protein and overall kJ intake and gestation at delivery also needs to be explored. The finding of significantly reduced gestational weight gain in women consuming a LpCD at 28 weeks’ gestation also warrants further investigation as this may be a therapeutic option for women at risk of significant weight gain in pregnancy. In addition, our study only examined women who were overweight or obese. It is therefore important to replicate the study in women of all BMI groups to determine if the same findings are seen in women in other BMI groups. 

Weaknesses include the fact that this study was observational and there may be unidentified confounders. Dietary data were self-reported in a questionnaire meaning there is a risk of recall bias as well as potential for underreporting that was not accounted for through removal of outliers. The overall daily kJ intake reported by some women, particularly those in the low absolute carbohydrate quintile, was low and not at a level known to typically meet pregnancy requirements. The Australian Government National Health and Medical Research Council recommends an energy intake of approximately 7000 kJ per day in pregnancy to meet basal metabolic requirements alone [17]. The mean energy intake in women consuming a low absolute carbohydrate diet was suggestive of under-reporting at 4300–4400 kJ/d. Another limitation of our study is that serum ketone levels were not measured, and it is unclear if women consuming a LaCD had increased ketogenesis. 

## 6. Conclusions

This study is the first to evaluate obstetric outcomes in overweight and obese women consuming a diet with low absolute carbohydrate intake during pregnancy. Consumption of a low absolute carbohydrate diet but not a low percentage carbohydrate diet is associated with increased gestation at delivery. It is unclear if a low absolute carbohydrate intake has long-term effects on the health of the infant. 

## Figures and Tables

**Table 1:** Patient demographics for women consuming a diet with carbohydrate intake in the lowest quintile in g/d vs. all other women.

Patient Characteristics	LaCD 16 (Q1) *N* = 79	SD 16 (Q2–5) *N* = 314	LaCD 28 (Q1) *N* = 76	SD 28 (Q2–5) *N* = 306	LaCD 16&28 (Q1) *N* ^1^ = 42	SD 16&28 (Q2–5) *N* = 266
Age, year	32 (7)	32 (5)	32 (5)	32 (5)	32 (5)	32 (5)
BMI, kg/m^2^	33 (6)	32 (6)	34 (6)	32 (6) *	33 (5)	32 (6)
Ethnicity						
Caucasian, n(%)	69 (87)	275 (88)	67 (88)	264 (86)	37 (88)	232 (87)
Asian, n(%)	2 (3)	12 (4)	3 (4)	12 (4)	1 (2)	10 (4)
Other, n(%)	8 (10)	27 (9)	6 (8)	30 (10)	4 (10)	24 (9)
Parity						
P0, n(%)	39 (49)	166 (53)	42 (55)	164 (54)	19 (45)	142 (53)
P1, n(%)	31 (39)	94 (30)	26 (34)	90 (29)	18 (43)	76 (29)
P2, n(%)	9 (11)	29 (9)	6 (8)	32 (10)	5 (12)	28 (11)
P3+, n(%)	0 (0)	25 (8)	2 (3)	20 (7)	0 (0)	20 (8)
Probiotic Use Y, n(%)	ND	ND	34 (45)	155 (51)	22 (52)	138 (52)
FHx Diabetes, n(%)	23 (29)	84 (27)	18 (24)	85 (28)	11 (26)	72 (27)
Previous GDM, n(%)	4 (5)	17 (5)	3 (4)	16 (5)	2 (5)	15 (6)

Data presented as means and standard deviation or n (%), * significant difference between groups (*p* < 0.05). ^1^ *N* is reduced in this group as women were required to be in Q1 at both time points, LaCD: Low absolute carbohydrate diet (diet with carbohydrate intake in the lowest quintile in g/d), SD: Standard diet (diet with carbohydrate intake in quintiles 2–5 in g/d), 16: 16 weeks, 28: 28 weeks, FHx Diabetes: Family history of diabetes mellitus. ND: Not determined.

**Table 2:** Dietary intake and metabolic parameters for women consuming a diet with carbohydrate intake in the lowest quintile in g/d vs. all other women.

Dietary Intake	LaCD 16 (Q1) *N* = 79	SD 16 (Q2–5) *N* = 314	LaCD 28 (Q1) *N* = 76	SD 28 (Q2–5) *N* = 306	LaCD 16&28 (Q1) *N* = 42 16 week	SD 16&28 (Q2–5) *N* = 266 16 week	LaCD 16&28 (Q1) *N* = 42 28 week	SD 16&28 (Q2–5) *N* = 266 28 week
CHO (g)	99 (14)	183 (48) *	101 (14)	183 (49) *	96 (12)	190 (48) *	100 (16)	187 (48) *
CHO (range, g)	(69–121)	(121–424)	(74–123)	(123–335)	(70–118)	(121–424)	(74–123)	(124–335)
*%CHO	39 (7)	43 (5) *	40 (6)	43 (5) *	39 (7)	43 (5) *	39 (6)	43 (5) *
LpCD, n (%)	36 (46)	43 (17) *	26 (34)	50 (16) *	19 (45)	23 (9) *	19 (45)	22 (8*)
Fat (g)	49 (16)	77 (26) *	47 (12)	78 (25) *	48 (17)	79 (26) *	48 (12)	79 (25) *
%Fat	41 (5)	39 (5) *	41 (5)	39 (5) *	41 (6)	39 (5) *	41 (5)	39 (5) *
Sat fat (g)	21 (7)	33 (12) *	21 (6)	34 (12) *	12 (7)	34 (12) *	12 (8)	34 (12) *
Protein (g)	58 (19)	87 (27) *	53 (15)	86 (27) *	58 (21)	89 (27) *	54 (14)	87 (26) *
% Protein	21 (4)	20 (3) *	20 (3)	19 (3) *	22 (3)	20 (3) *	20 (3)	19 (5) *
Junk food (g)	35 (24)	79 (51) *	38 (19)	81 (48) *	35 (19)	83 (53) *	39 (20)	83 (47) *
Total kJ	4447 (983)	7392 (2032) *	4341 (758)	7401 (2023) *	4384 (1037)	7607 (2040) *	4375 (749)	7513 (1996) *
FBG	4.4 (0.4)	4.4 (0.4)	4.3 (0.5)	4.4 (0.5)	4.3 (0.4)	4.4 (0.4)	4.4 (0.4)	4.4 (0.5)
C-peptide	0.6 (0.3)	0.6 (0.2)	0.8 (0.3)	0.9 (0.4)	0.5 (0.2)	0.6 (0.2)	0.7 (0.3)	0.8 (0.4) *
Insulin	8.0 (4.6)	7.4 (4.6)	9.8 (5.3)	10.6 (9.2)	7.4 (4.5)	7.4 (4.7)	9.8 (5.3)	10.6 (9.7)
Triglyceride	1.4 (0.7)	1.5 (0.5)	2.2 (0.7)	2.2 (0.8)	1.4 (0.6)	1.5 (0.6)	2.1 (0.7)	2.2 (0.7)
Cholesterol	5.5 (0.9)	5.5 (0.9)	6.9 (1.2)	6.6 (1.1)	5.6 (1.0)	5.5 (1.0)	6.8 (1.1)	6.6 (1.1)
HOMA-IR	1.6 (1.0)	1.5 (1.0)	1.9 (1.1)	2.2 (2.5)	1.5 (1.0)	1.5 (1.0)	2.0 (1.2)	2.2 (2.6)
Fibre (g)	13 (3)	20 (6) *	11 (3)	20 (6) *	12 (3)	21 (6) *	12 (3)	20 (6) *
Folate (mcg)	160 (42)	252 (76) *	147 (36)	246 (73) *	160 (40)	260 (77) *	150 (36)	258 (76) *
Thiamine (mg)	0.8 (0.2)	1.5 (0.5) *	0.8 (0.2)	1.4 (0.5) *	0.8 (0.2)	1.5 (0.5) *	0.8 (0.2)	1.5 (0.5) *

Data are presented as means and standard deviation * Significant difference between groups at each timepoint, *p* < 0.05, LaCD: Low absolute carbohydrate diet (diet with carbohydrate intake in the lowest quintile in g/d), SD: Standard diet (diet with carbohydrate intake in quintiles 2–5 in g/d), LpCD: Low percentage carbohydrate diet (diet with carbohydrate intake in the lowest quintile when measured as a percentage of overall kilojoule intake), 16: 16 weeks, 28: 28 weeks, CHO: Carbohydrate, Sat fat: saturated fat.

**Table 3:** Obstetric outcomes for women consuming a diet with carbohydrate intake in the lowest quintile in g/d vs. all other women.

Outcome	LaCD 16 (Q1) *N* = 79	SD 16 (Q2–5) *N* = 314	*p*	LaCD 28 (Q1) *N* = 76	SD 28 (Q2–5) *N* = 306	*p*	LaCD 16 & 28 (Q1) *N* = 42	SD 16 & 28 (Q2–5) *N* = 266	*p*
Birth centile ^1^	46 (29)	52 (30)	0.08	45 (29)	53 (29)	0.04 *	43 (29)	53 (30)	0.04 *
Birth weight (g) ^1^	3575 (401)	3524 (558)	0.36	3528 (464)	3538 (540)	0.88	3566 (414)	3528 (557)	0.60
Birth weight z score ^1^	0.4 (1.1)	0.6 (1.3)	0.31	0.3 (1.2)	0.6 (1.3)	0.08	0.3 (1.2)	0.6 (1.3)	0.22
Pregnancy weight gain (kg) ^1^	7.7 (5.6)	7.8 (5.6)	0.88	7.7 (4.5)	8.1 (5.8)	0.42	7.4 (5.2)	8.0 (5.8)	0.53
Gestational age (weeks) ^1^	39.7 (1.3)	39.1 (1.9)	0.008 *	39.5 (1.6)	39.2 (1.9)	0.20	39.8 (1.2)	39.1 (1.9)	0.005 *
Pre-term (34) (<34 weeks) n(%)	0 ^6^ (0)	8 ^30^ (3)	0.37	1 ^5^ (1)	7 ^31^ (2)	1.0	0 ^1^ (0)	7 ^26^ (3)	0.60
Pre-term (37) (<37 weeks) n(%)	2 ^6^ (3)	25 ^30^ (8)	0.09	4 ^5^ (5)	22 ^31^ (7)	0.62	1 ^1^ (2)	21 ^26^ (8)	0.22
Full-term (≥40 weeks) n(%)	34 ^3^ (43)	103 ^30^ (33)	0.14	31 ^5^ (41)	103 ^31^ (34)	0.34	21 (50) ^1^	88 (33) ^26^	0.08
Female infant sex, n(%)	42 (53)	157 (50)	0.71	37 (49)	156 (41)	0.80	19 (45)	132 (50)	0.62
LBW, n(%)	0 (0)	13 (4)	0.08	1 ^1^ (1)	11 (4)	0.47	0 (0)	11 (4)	0.37
Macrosomia, n(%)	12 (15)	51 (16)	1.0	10 ^1^ (13)	51 (17)	0.60	5 (12)	42 (16)	0.65
Neonatal hypoglycaemia, n(%)	7 ^1^ (9)	44 ^7^ (14)	0.26	12 ^1^ (16)	38 ^7^ (12)	0.45	4 (10)	35 (13) ^6^	0.62
ICN admission, n(%)	12 ^3^ (15)	69 ^2^ (22)	0.27	19 ^2^ (25)	58 ^3^ (19)	0.26	7 (17) ^1^	53 (20) ^1^	0.83
Jaundice, n(%)	14 ^1^ (18)	59 ^4^ (19)	1.0	11 ^1^ (14)	58 ^3^ (19)	0.50	7 (17) ^1^	51 (19) ^3^	0.83
Respiratory difficulties, n(%)	10 ^1^ (13)	42 ^8^ (13)	1.0	13 ^2^ (17)	36 ^3^ (12)	0.25	4 (10) ^1^	30 (11) ^3^	1.0
IOL or C-section, n(%)	44 (56)	196 ^2^ (62)	0.25	46 ^1^ (61)	189 ^1^ (62)	0.92	26 (62)	170 (64) ^1^	0.86
PET, n(%)	6 (8)	32 ^1^ (10)	0.67	10 ^1^ (13)	27 (9)	0.28	4 (10)	26 (10)	1.0
HDP, n(%)	12 (15)	46 ^1^ (15)	0.86	13 ^1^ (17)	43 (14)	0.47	6 (14)	39 (15)	1.0
GDM, n(%)	11 (14)	51 (16)	0.73	10 (13)	49 (16)	0.60	4 (10)	43 (16)	0.36

* significant difference between groups (*p* < 0.05), ^1^ mean (SD), LaCD: Low absolute carbohydrate diet (diet with carbohydrate intake in the lowest quintile in g/d), SD: Standard diet (diet with carbohydrate intake in quintiles 2–5 in g/d), 16: 16 weeks, 28: 28 weeks, ICN: Intensive care nursery, PET: Preeclampsia, HDP: Hypertensive disorder of pregnancy, Numeric superscript indicates number of patients for whom data are missing.

**Table 4:** Association between total kJ, fat and protein intake and gestation at delivery and birth centile.

	Intake Range		Gestation at Delivery (weeks)		Birth Centile	
Dietary Intake	16 weeks	28 weeks	16: Q1 vs. Q2–5	28: Q1 vs. Q2–5	16: Q1 vs. Q 2–5	28: Q1 vs. Q2–5
Total kJ/d	Q1: 2130–8305 Q2–5: 3975–17,009	Q1: 2506–6949 Q2–5: 3943–14,119	39.6 vs. 39.1 *p* = 0.009 *	39.6 vs. 39.2 *p* = 0.07	46th vs. 52nd *p* = 0.10	46th vs. 52nd *p* = 0.13
Protein (g/d)	Q1: 20–148 Q2–5: 36–208	Q1: 27–111 Q2–5: 35–204	39.6 vs. 39.1 *p* = 0.02 *	39.5 vs. 39.2 *p* = 0.25	46th vs. 52nd *p* = 0.14	48th vs. 52nd *p* = 0.35
Fat (g/d)	Q1: 17–116 Q2–5: 33–194	Q1: 20–89 Q2–5: 60–175	39.5 vs. 39.2 *p* = 0.15	39.5 vs. 39.2 *p* = 0.13	48th vs. 52nd *p* = 0.34	48th vs. 52nd *p* = 0.37

* significant difference between groups (*p* < 0.05), 16: 16 weeks, 28: 28 weeks.

**Table 5:** Obstetric outcomes for women consuming a diet with carbohydrate content of less than 175 g/d vs. greater than 175 g/d.

Outcome	<175 g (16) *N* = 155	≥175 g (16) *N* = 238	*p*	<175 g (28) *N* = 134	≥175 g (28) *N* = 248	*p*
Birth centile ^1^	54 (30)	49 (29)	0.11	53 (28)	50 (30)	0.42
Birth weight (g) ^1^	3538 (539)	3532 (526)	0.91	3552 (473)	3528 (553)	0.65
Birth weight z score ^1^	0.6 (1.3)	0.5 (1.2)	0.37	0.6 (1.1)	0.5 (1.3)	0.80
Pregnancy weight gain (kg) ^1^	8.3 (6.2)	7.4 (5.2)	0.16	8.3 (5.2)	7.8 (5.7)	0.38
Gestational age (weeks) ^1^	39.1 (1.9)	39.3 (1.8)	0.36	39.3 (1.6)	39.2 (1.9)	0.69
Very pre-term (<34 weeks) n(%)	4 ^18^ (3)	4 ^18^ (2)	0.49	2 ^13^ (1)	6 ^23^ (2)	0.72
Pre-term (<37 weeks) n(%)	11 ^18^ (7)	16 ^18^ (7)	0.84	6 ^13^ (4)	20 ^23^ (8)	0.21
Full-term (≥40 weeks) n(%)	47 ^18^ (30)	90 ^18^ (38)	0.22	44 ^13^ (33)	90 ^23^ (36)	0.56
Female infant sex, n(%)	69 (45)	130 (55)	0.06	61 (46)	132 (53)	0.16
LBW, n(%)	4 (3)	9 (4)	0.59	4 (3)	8^1^ (3)	1.0
Macrosomia, n(%)	24 (15)	39 (16)	0.89	16 (12)	45 ^1^ (18)	0.14
Neonatal hypoglycaemia, n(%)	17 ^3^ (11)	34 ^5^ (14)	0.36	12 ^2^ (9)	38 ^6^ (15)	0.08
ICN admission, n(%)	28 ^1^ (18)	53 ^4^ (22)	0.31	23 (17)	54 ^5^ (22)	0.29
Jaundice, n(%)	27 ^2^ (17)	46 ^3^ (19)	0.69	16 ^1^ (12)	53 ^4^ (21)	0.03 *
Respiratory difficulties, n(%)	17 ^2^ (11)	35 ^4^ (15)	0.29	13 ^1^ (10)	16 ^4^ (6)	0.20
IOL or C-section, n(%)	93 (60)	147 (62)	0.67	71 (53)	164 ^2^ (66)	0.01 *
PET, n(%)	13 (8)	25 ^1^ (11)	0.60	10 (7)	27 ^1^ (11)	0.37
HDP, n(%)	18 (12)	40 ^1^ (17)	0.19	13 (10)	43 ^1^ (17)	0.048 *
GDM, n(%)	26 (17)	36 (15)	0.67	18 (13)	41 (17)	0.46

* significant difference between groups (*p* < 0.05), ^1^ mean (SD), Numeric superscript indicates number of patients for whom data are missing, 16: 16 weeks, 28: 28 weeks.

## Data Availability

Data are available upon request from corresponding author.

## Supplementary Materials

The following are available online at https://www.mdpi.com/article/10.3390/nu13103511/s1, Table S1: Patient demographics for women consuming a diet with carbohydrate in-take in the lowest quintile measured as a percentage of overall kilojoule intake vs all other women, Table S2: Dietary intake and metabolic parameters for women consuming a diet with carbohydrate intake in the lowest quintile measured as a percentage of overall kilojoule in-take vs all other women, Table S3: Obstetric outcomes for women consuming a diet with carbohydrate intake in the lowest quintile measured as a percentage of overall kilojoule intake vs all other women.

## References

[1] Major C.A., Henry M.J., De Veciana M., Morgan M.A. (1998). The effects of carbohydrate restriction in patients with diet-controlled gestational diabetes. Obstet. Gynecol..

[2] Moreno-Castilla C., Hernandez M., Bergua M., Alvarez M.C., Arce M.A., Rodriguez K., Martinez-Alonso M., Iglesias M., Mateu M., Santos M.D. (2013). Low-carbohydrate diet for the treatment of gestational diabetes mellitus: A randomized controlled trial. Diabetes Care.

[3] Cypryk K., Kaminska P., Kosinski M., Pertynska-Marczewska M., Lewinski A. (2007). A comparison of the effectiveness, tolerability and safety of high and low carbohydrate diets in women with gestational diabetes. Endokrynol. Pol..

[4] Adam-Perrot A., Clifton P., Brouns F. (2006). Low-carbohydrate diets: Nutritional and physiological aspects. Obes. Rev..

[5] Metzger B.E., Ravnikar V., Vileisis R.A., Freinkel N. (1982). “Accelerated starvation” and the skipped breakfast in late normal pregnancy. Lancet.

[6] American Diabetes A. (2018). 13. Management of Diabetes in Pregnancy: Standards of Medical Care in Diabetes-2018. Diabetes Care.

[7] Mijatovic J., Louie J.C.Y., Buso M.E.C., Atkinson F.S., Ross G.P., Markovic T.P., Brand-Miller J.C. (2020). Effects of a modestly lower carbohydrate diet in gestational diabetes: A randomized controlled trial. Am. J. Clin. Nutr..

[8] Callaway L.K., McIntyre H.D., Barrett H.L., Foxcroft K., Tremellen A., Lingwood B.E., Tobin J.M., Wilkinson S., Kothari A., Morrison M. (2019). Probiotics for the Prevention of Gestational Diabetes Mellitus in Overweight and Obese Women: Findings From the SPRING Double-Blind Randomized Controlled Trial. Diabetes Care.

[9] Giles CG I.P. (1996). Dietary Questionnaire for Epidemiological Studies (Version 2) Australia. Cancer Counc. Aust..

[10] Hodge A., Patterson A.J., Brown W.J., Ireland P., Giles G. (2000). The Anti Cancer Council of Victoria FFQ: Relative validity of nutrient intakes compared with weighed food records in young to middle-aged women in a study of iron supplementation. Australian N. Z. J. Public Health.

[11] Boylan S., Hardy L.L., Drayton B.A., Grunseit A., Mihrshahi S. (2017). Assessing junk food consumption among Australian children: Trends and associated characteristics from a cross-sectional study. BMC Public Health.

[12] Lowe S.A., Bowyer L., Lust K., McMahon L.P., Morton M.R., North R.A., Paech M.J., Said J.M. (2015). The SOMANZ Guidelines for the Management of Hypertensive Disorders of Pregnancy 2014. Australian. N. Z. J. Obstet. Gynaecol..

[13] Metzger B.E., Gabbe S.G., Persson B., Buchanan T.A., Catalano P.A., Damm P., Dyer A.R., Leiva A., Hod M., Kitzmiler J.L. (2010). International association of diabetes and pregnancy study groups recommendations on the diagnosis and classification of hyperglycemia in pregnancy. Diabetes Care.

[14] Joseph F.A., Hyett J.A., Schluter P.J., McLennan A., Gordon A., Chambers G.M., Hilder L., Choi S.K., de Vries B. (2020). New Australian birthweight centiles. Med. J. Aust..

[15] Najpaverova S., Kovarik M., Kacerovsky M., Zadak Z., Hronek M. (2020). The Relationship of Nutritional Energy and Macronutrient Intake with Pregnancy Outcomes in Czech Pregnant Women. Nutrients.

[16] Patel M.S., Johnson C.A., Rajan R., Owen O.E. (1975). The metabolism of ketone bodies in developing human brain: Development of ketone-body-utilizing enzymes and ketone bodies as precursors for lipid synthesis. J. Neurochem..

[17] Australian Government, National Health and Medical Research Council Nutrient Reference Values for Australia and New Zealand. https://www.nrv.gov.au/nutrients/folate.

[18] Australian Bureau of Statistics Australian Health Survey: Nutrition First Results—Foods and Nutrients, 2011–2012 Financial Year Results. https://www.abs.gov.au/statistics/health/health-conditions-and-risks/australian-health-survey-nutrition-first-results-foods-and-nutrients/2011-12#energy-and-nutrients.

[19] Food Standards Australia and New Zealand Folic Acid/Folate and Pregnancy. https://www.foodstandards.gov.au/consumer/generalissues/pregnancy/folic/Pages/default.aspx.

[20] Gomez-Arango L.F., Barrett H.L., Wilkinson S.A., Callaway L.K., McIntyre H.D., Morrison M., Dekker Nitert M. (2018). Low dietary fiber intake increases Collinsella abundance in the gut microbiota of overweight and obese pregnant women. Gut Microbes.

[21] Tapsell L. (2019). Food, Nutrition and Health.

[22] MacKay D.F., Smith G.C., Dobbie R., Pell J.P. (2010). Gestational age at delivery and special educational need: Retrospective cohort study of 407,503 schoolchildren. PLoS Med..

[23] Murray S.R., Shenkin S.D., McIntosh K., Lim J., Grove B., Pell J.P., Norman J.E., Stock S.J. (2017). Long term cognitive outcomes of early term (37–38 weeks) and late preterm (34–36 weeks) births: A systematic review. Wellcome Open Res..

[24] Ahlsson F., Kaijser M., Adami J., Lundgren M., Palme M. (2015). School performance after preterm birth. Epidemiology.

[25] Tanner H.L., Dekker Nitert M., Callaway L.K., Barrett H.L. (2021). Ketones in Pregnancy: Why Is It Considered Necessary to Avoid Them and What Is the Evidence Behind Their Perceived Risk?. Diabetes Care.

