# Supplementary material for: Consumption of a Low Carbohydrate Diet in Overweight or Obese Pregnant Women Is Associated with Longer Gestation of Pregnancy
Source: Nutrients. 2021 Oct 5;13(10):3511. doi: 10.3390/nu13103511 (PMC8538994; doi:10.3390/nu13103511)
Supplement: Supplementary file 1 [file nutrients-13-03511-s001.zip › nutrients-1371172-supplementary.pdf]

The editors' comments to the authors:

This manuscript presents very interesting data on a low carbohydrate diet in pregnancy.
However, there are some concerns about the over interpretation of the regression analysis
data and the results derived.
Authors should describe the limitations and the interpretations carefully.

Thank you for your comments.

1) The authors showed that a low absolute carbohydrate intake at 16 and at 28 weeks'
gestation was associated with increased gestation at delivery (16: $p = 0.04$, adjusted $R2 = 0.15$, 28: $p = 0.04$, adjusted $R2 = 0.20$) in linear regression analysis.

The authors describe it as logistic regression in the paper. (11of 22)
If the gestation at delivery was a continuous value, did the authors perform a multiple
regression analysis instead of a logistic regression?
The authors should clarify it and show the standardized regression coefficient of beta (β)
to indicate the effect of the independent variable.

We performed a linear regression rather than logistic regression as per the recommendations from reviewers. Logistic regression was meant to have been removed from the manuscript that was received on the 22nd of September, however it appears it was still present in the manuscript currently in the system. We apologise for any confusion with the multiple versions of the manuscript. We have added the Coefficient of beta for consumption of a LaCD in the gestation at delivery analysis.

2) The adjusted R2 for the models were 0.15 and 0.20. These values indicate that
the model does not explain much of the variance in the dependent variable.
The criteria are not clear to select the variables for adjustment. The model may not
be adjusted for potential confounding factors.
The authors should discuss the limitations and make the authors' conclusion using
this model carefully.

We have adjusted for confounders in the regression analysis using a forward step-wise regression approach. We have added the word multivariate to the following paragraph in the method's section:

To control for the effect of confounders on length of gestation and birth centile in women consuming a LaCD, linear regression was performed. The following demographic and

obstetric factors were initially examined in a univariate analysis: maternal age at delivery, parity, BMI, ethnicity, probiotic use, family history of diabetes, GDM status, IOL and/or C-section, hypertensive disorder of pregnancy, infant sex and weight gain during the pregnancy. All factors that reached a significance level of $p<0.1$ were entered into a <u>*multivariate*</u> *linear regression model. Results were considered statistically significant if $p < 0.05$.*

We have also now changed the results section to more clearly highlight the adjustment for confounders as per below and included the coefficient of beta.

Linear regression

The following factors were found to be associated with gestation at delivery when performing univariate analysis using a $p < 0.1$; BMI, previous GDM, infant sex, GDM, HDP, IOL or C-section and gestational weight gain. After adjusting for these factors, consumption of a LaCD at 16 and at 28 weeks' gestation was significantly associated with increased gestation at delivery when analysed via linear regression: (16: $p = 0.04$, adjusted $R^2 = 0.15$, 28: $p = 0.04$, adjusted $R^2 = 0.17$). The coefficient of beta for consumption of a LaCD at 16 weeks' gestation was 0.50 (95%CI 0.03 – 0.98) and at 28 weeks' gestation was 0.51 (95%CI 0.03 – 0.99) meaning that consumption of a LaCD accounted for an extra 3.5 days in gestational age. All other factors that were significantly associated with gestation in the model reduced the gestational age at delivery.

The following factors were found to be associated with birth centile when performing univariate analysis using a $p < 0.1$; family history of diabetes and gestational weight gain. After adjusting for these factors, there was a trend to an association between the consumption of a LaCD at 16 weeks and at 28 weeks' gestation and birth centile (16: $p = 0.08$, adjusted $R^2 = 0.02$, 28: $p = 0.07$ adjusted $R^2 = 0.02$).

We have added a sentence to the discussion regarding the R^2 values as per below:

The adjusted R^2 value for the association between a LaCD and gestation at delivery was 0.15 at 16 weeks' and 0.17 at 28 weeks' gestation, indicating that consumption of a LaCD explained 15-17% of the variability in gestational age at delivery. The results for the coefficient of beta showed that consumption of a LaCD accounted for an additional 3.5 days in gestational age at delivery.

3) My main scientific concern is that the data are greatly over-interpreted.
I wonder
if the results have any clinically significant value.
Do the authors assume that increased gestation at delivery is due to a delay
in the
natural onset of labor?

If there is a possibility to include cases of artificial induction of labor, the limitations should also be stated.

We have adjusted for IOL and C-section in the linear regression model and the association between a low carbohydrate diet and increased gestation remains. A possibility is that there is a delay in the natural onset of labour. It is unclear why this would be, however the same results have been found in a previous study which is discussed in the discussion. There is some data to suggest that childhood IQ and school performance are affected by gestation at delivery, even at the latter end of pregnancy, suggesting the results could have clinically significant value. This is discussed in the discussion. We have concluded based on the statistical analysis that there is an association between carbohydrate intake and gestational age. We think that this is an appropriate interpretation of the results even though it is an unexpected finding.

We have changed the title to the following which we feel appropriately represents the findings.

Consumption of a low carbohydrate diet in overweight or obese pregnant women is associated with longer gestation of pregnancy
